# Transdiagnostic Psychological Interventions for Symptoms of Common Mental Disorders Delivered by Non-Specialist Providers in Low- and Middle-Income Countries: A Systematic Review and Meta-Analysis

**DOI:** 10.1155/2024/5037662

**Published:** 2024-07-10

**Authors:** Éanna Ó hAnrachtaigh, Gary Brown, Andrew Beck, Rebecca Conway, Hattie Jones, Ioannis Angelakis

**Affiliations:** ^1^Royal Holloway, University of London, London, UK; ^2^University of Hertfordshire, Hatfield, UK; ^3^Bradford Teaching Hospitals NHS Foundation Trust, Bradford, UK; ^4^Berkshire NHS Foundation Trust, Berkshire, UK; ^5^University of Liverpool, Liverpool, UK

## Abstract

There is a treatment gap for Common Mental Disorders (CMDs) such as anxiety, depression, and post-traumatic stress disorder (PTSD), as well as non-specific psychological distress (NPD) in low- and middle-income countries (LAMIC), due to the lack of available clinicians and locally appropriate interventions. Task-shifting using non-specialist providers (NSP) and transdiagnostic approaches may address this. Transdiagnostic approaches can be effective at treating CMDs and NPD in high-income countries (HIC), but currently, there is no comprehensive synthesis of evidence regarding their effectiveness in LAMICs. This review addressed this gap by examining the effectiveness of transdiagnostic psychological interventions for symptoms of CMDs and NPD delivered by NSPs in LAMICs. Three databases were searched (Embase, PsycInfo, and PubMed). Hedge's *g*'s were calculated using random-effect models to correct for small sample bias. Twenty-one transdiagnostic interventions across 20 studies were included. Moderate reductions at posttreatment were found in psychological distress (g = −0.64; 95% CI: −0.81 to −0.46), symptoms of anxiety (g = −0.61; 95% CI: −0.80 to −0.42), depression (g = −0.59; 95% CI: −0.75 to −0.44), and PTSD/trauma (g = −0.38; 95% CI: −0.59 to −0.16), with significant small reductions maintained at follow-up ranging from 13 to 26 weeks for NPD (SMD = − 0.37; 95% CI: −0.57 to −0.17), anxiety (g = − 0.41; 95% CI: −0.91 to 0.09), depression (g = −0.38; 95% CI: −0.59 to −0.16), and trauma symptoms (g = −0.23; 95% CI: −0.42 to −0.05). These findings are important and suggest that transdiagnostic approaches delivered by NSPs are effective at treating symptoms of CMDs and NPD in LAMICs. Future research should consider comparing task-shifting approaches with disorder-specific interventions and explore the effectiveness of longer sessions across various mental health conditions.

## 1. Introduction

Common Mental Disorders (CMDs) comprise a range of mental health categories, including generalized anxiety disorder (GAD), social anxiety disorder, obsessive-compulsive disorder (OCD), panic disorders, specific phobias, post-traumatic stress disorder (PTSD), and major depressive disorder (MDD) or dysthymia [1]. CMDs constitute a global problem, with 264–322 million people worldwide affected by depression [2], 227 million by PTSD [3], and approximately 284 million people affected by anxiety [4]. Studies have also consistently shown that CMDs are underdiagnosed and underreported [5, 6], and there is evidence of up to 31% of populations reporting nonspecific psychological distress (NPD) [7]. Rates of CMDs and NPD continue to rise internationally [8] and pose a significant economic burden estimated to be as high as US$ 1.15 trillion a year [9]. These costs are associated with losses in productivity in the workplace, medical costs, and suicide-related expenses [10]. Despite the high burden of CMDs and NPD, a global “treatment gap” exists between the number of people experiencing mental health difficulties and the number that are accessing appropriate mental health services [11]. For example, rates for untreated depression range from 51.7% to 83.2% [12] and 72.4% for anxiety disorders [13]. While this treatment gap has been shown to be universal [14], the size of this gap differs substantially between high-income countries (HICs), defined by the World Bank as economies with a gross national income (GNI) of $12,695 or more in 2020 [15], and low- and middle-income countries (LAMICs) with GNI of $12,695 or less in 2020. While LAMICs are currently home to 83% of the global population [16], individuals in these countries report lower level of access to mental health services than HICs; a higher rate of CMD remaining undiagnosed [17]; and a lower number of individuals treated “effectively” [18]. A substantial factor in this treatment gap is a deficit in resources; the World Mental Health Atlas estimates 2.7 psychologists per 1,000,000 population in HICs, compared to 0.2 psychologists per 1,000,000 in LAMICs [19]. This highlights the urgent need to address the lack of adequate and accessible health care within LAMICs.

Given the estimated requirement for an additional 1.71 million mental health workers in LAMICs over a 10-year period to address the gap in services [20], task-shifting is seen as an important tool in addressing the shortage of trained mental health professionals [21]. This requires shifting tasks among professional health workers to trained members of the community [22]. It involves the delegation of specific service delivery tasks from individuals with professional qualifications to non-specialist providers (NSPs) with no formal qualifications or training in mental health care provision [23]. NSPs have increasingly been used in the delivery of interventions within LAMIC to address the treatment gap [24]. NSPs refer to a broad range of providers, outlined in Table 1 below, including professionals such as doctors, teachers, nurses, or pharmacists, to paraprofessionals such as community workers, volunteers, or lay persons such as peers or refugees [25], that can deliver brief, low-intensity psychological interventions following minimal training, often under the supervision of specialist mental health professionals [26].

Given the high level of comorbidity within CMDs, transdiagnostic approaches can be an important tool in reducing the burden of task-shifting in LAMICs [27]. Instead of being trained in several different interventions, NSPs can instead be trained in one approach that can then be applied across a range of conditions. This growing interest in transdiagnostic solutions has led to the development of novel transdiagnostic approaches, such as Problem Management Plus (PM+) [28] based on components of Mental Health Gap Action Programme Intervention Guide [29] or Common Elements Treatment Approach (CETA) [30]. There remains some uncertainty about what constitutes a “transdiagnostic intervention”. Gutner et al., [31] defined transdiagnostic treatments as any intervention designed to “specifically target psychological processes or core vulnerabilities that have been observed to contribute to the development and maintenance of classes of disorders” (p. 2). Others [32] have suggested that transdiagnostic approaches can fall into three broad categories – (1) Universally Applied Therapeutic Principles, (2) Modular Treatments, and (3) Shared Mechanism Treatments, with this subsequently expanded [33] to include (4) principle-guided approaches (see Table 2, below).

Many of these transdiagnostic approaches share cognitive behavioral elements such as behavioral activation and thought restructuring; the structured approaches used in Cognitive Behavioural Therapy (CBT)-based interventions may be well suited for task-shifting to NSPs [34]. Although there is strong evidence for transdiagnostic treatments within HIC [35, 36 and 37], the effectiveness of these approaches for CMDs and NPD within LAMICs remains unclear. Schäfer et al. [38] recently completed a review of transdiagnostic psychological interventions for forcibly displaced people, but it did not focus on those delivered by NSPs and focused on preventative interventions. Furthermore, Murray et al. [27] (2019) narrative synthesis of transdiagnostic approaches within LAMICs outlined existing approaches but did not examine the effectiveness of these treatments.

To address this important gap, we examined the effectiveness of transdiagnostic psychological treatments for symptoms of CMDs in LAMICs. In accordance with the increasing significance of task-shifting within service delivery, our review focused on studies that are delivered exclusively by NSPs. We also restricted our searches to cognitive–behavioural-based treatments because these approaches are likely to be more structured and manualized, and more suitable for task-shifting. Given the higher rate of under diagnosis of CMDs and the reduced access to health professionals to allow formal diagnosis in LAMIC, the review focused on those with both symptoms of CMDs and NPD. There were two core aims:

1. To evaluate the effectiveness of transdiagnostic psychological interventions for CMDs in LAMICs on psychological distress, anxiety, depression, and trauma symptoms,
2. To explore if the effectiveness of transdiagnostic intervention is affected by methodological, participant, geographical, and intervention characteristics.

## 2. Methods

### 2.1. Protocol and Registration

The systematic review and meta-analysis were conducted in accordance with Preferred Reporting Items for Systematic Reviews and Meta-Analysis guidelines (PRISMA) [39], included in Table 3. The protocol was registered prior to the outset of the review with PROSPERO on 9^th^ August 2021 (Prospero ID: CRD42021267519).

### 2.2. Patient and Public Involvement

It was not possible to involve patients or the public in the design or conduct of the review.

### 2.3. Criteria for Evaluating Studies

#### 2.3.1. Inclusion Criteria

*(1) Participants*. Studies recruiting adults (18 years old or over) living in a LAMIC (based on World Bank classifications) i) reporting NPD or symptoms of anxiety, trauma, and/or depression, ii) a diagnosis of an anxiety disorder, depressive disorder, or a mixed anxiety and depression disorder, or iii) seeking treatment for anxiety and/or depression. The DSM-IV [40] categorizations of anxiety disorders were used, including PTSD to capture the World Health Organisations definition of CMD [2].

*(2) Interventions*. Studies focusing on manualised transdiagnostic cognitive behavioral-based interventions, delivered by a NSP specifically targeting i) two or more anxiety disorders/symptoms or ii) both an anxiety and depressive disorder/symptoms. NSP's were defined as any individual providing mental healthcare without having received specialized training in mental health [41]. Interventions must have been based on CBT, including third-wave approaches such as Acceptance and Commitment Therapy (ACT) [42] or include substantial elements of cognitive behavioral techniques (exposure, behavioral experiment, behavioral activation, cognitive restructuring, etc.). Guided self-help interventions were included, provided a substantial part of the therapeutic intervention was delivered face-to-face by NSPs. Because of greater difficulties accessing internet in some LAMICs, digital interventions were excluded altogether. Due to the lack of consensus on what constitutes “transdiagnostic interventions,” treatments that specifically targeted more than one disorder were classed as transdiagnostic for the purposes of this review, even if the authors did not use this wording.

*(3) Research Design and Comparisons*. Studies utilizing randomized controlled trial methodology (including cluster randomization) in which a psychological intervention was compared to a control condition (enhanced treatment as usual, waiting list, etc.), another disorder-specific treatment, or a control psychological treatment. No limit was placed on publication date.

*(4) Outcomes*. Studies using a validated outcome measure of NPD (measures such as the General Health Questionnaire (GHQ-12) and Kessler 6), anxiety, depression, or trauma (self-report or clinician-rated) at least one-time point post intervention.

#### 2.3.2. Exclusion Criteria

Studies were excluded that (a) involved interventions that were not manualized, were solely self-help, or involved no direct face-to-face therapeutic contact with an NSP, (b) focused on populations under 18 years old (mean age of all participants had to be >18 years old), (c) included participants with comorbid severe mental illness (e.g., psychosis, personality disorders, etc.), (d) participants who were approached by researchers from the general population, were not seeking support for distress, or were not screened for distress or CMDs, (e) examined quasi-experimental designs and case studies, (e) compared two eligible transdiagnostic interventions, and (f) that were not published in English or that were not published in peer-reviewed journals.

### 2.4. Search Methods for Study Identification

#### 2.4.1. Electronic Searches

Embase, PsycInfo, and PubMed were all searched, and data were extracted on 12^th^ August 2021, with an updated search conducted on 8^th^ February 2023. Reference lists from previous systematic reviews and identified eligible studies were reviewed for additional potential studies. Search terms were compiled including suitable synonyms based on five main areas: (1) psychological interventions, (2) CMDs and NPD, (3) randomized controlled trials, (4) LAMICs, and (5) task shifting and NSPs (Table 4), with searches applied to title and abstracts and limited to English language and human studies. To capture transdiagnostic studies, psychological interventions that have been applied across numerous disorders were identified and listed, for example, CBT, ACT, mindfulness approaches, and modular treatments.

### 2.5. Study Selection

Following extraction from electronic databases, references were stored in Rayyan reference management software [43] where duplicates were identified and removed. The initial screening process was based on the guidance developed by Cochrane [44] with the first reviewer (ÉÓ) screenings title and abstract of all initial articles identified, and a second reviewer (RC or IA) screening 20% of studies independently, while all full texts were independently screened by two reviewers (ÉÓ and RC or HJ). Where conflicting judgments on inclusion arose, a third reviewer (GB) made the final decision on inclusion. There was a high level of agreement between reviewers for initial screening of titles and abstracts with inter-rater agreement of 98.75%, and a 92.9% level of initial agreement between raters for full-text screening.

### 2.6. Data Extraction and Management

Data were extracted to Microsoft Excel Spreadsheets, including (a) author name, (b) year of publication, (c) country in which the study was conducted, (d) design/unit of analysis, (e) recruitment, (f) participant age, (g) participant gender, (h) total sample size, (i) inclusion criteria, (j) intervention characteristics (format and structure, number and length of sessions), (k) control characteristics (enhanced treatment as usual [ETAU], waiting list, and other), (l) primary and secondary outcome measures, and (m) facilitators of interventions (education level and training). Means, standard deviations, and number of participants were extracted for all available outcome measures that met the criteria of the current review (distress, depression, anxiety, and trauma), at posttreatment and, where available, follow-up. To reduce error, data extraction was subsequently checked by a second coder. Where outcome measures were recorded at more than one time post intervention, the closest time point post intervention was selected to ensure maximum uniformity between included studies. Where studies reported data from more than one RCT, the data was treated as two separated trials, whereas if data for a single trial was published across multiple articles, the data were combined and considered as a single study.

### 2.7. Assessment of Risk of Bias in Included Studies

Risk of bias within the included studies was assessed using the Cochrane “Risk of Bias tool” [45]. This divided risk into seven different domains: (1) Random sequence generation (selection bias), (2) Allocation concealment (selection bias), (3) Blinding of participants and personnel (performance bias), (4) Blinding of outcome assessment (detection bias), (5) Incomplete outcome data (attrition bias), (6) Selective reporting (reporting bias), and (7) Other bias. Assessing these various domains, risk could be rated as “Low,” “Unclear,” or “High”, based on the information provided within selected studies. Study quality was assessed independently by two reviewers (ÉÓ and RC), and any conflicts were discussed between reviewers. Where these conflicting assessments were not resolved, a third reviewer (GB) made the final decision. There was initially 77.4% inter-rater reliability (IRR) between both quality assessors. Cohen's kappa score for IRR was 0.53 (95% CI 0.41, 0.65), indicating moderate agreement [46].

### 2.8. Data Synthesis

Standardized mean differences (SMDs) and 95% confidence intervals comparing group treatment effects between transdiagnostic interventions and control conditions at both posttreatment and follow-up were calculated in Stata 17 [47]. To account for small sample sizes within the included studies, and adjusted effect size, Hedge's *g* was used [48]. Based on previous reviews within this area, a high degree of heterogeneity among studies was expected. To account for it, pooled effect sizes were calculated using random effects models [49]. Effect sizes of 0.2, 0.5, and 0.8 were classed as small, medium, and large, respectively [50]. To test for the homogeneity of the selected studies, both the Q statistic and the *I*^2^ statistic were calculated, with scores of 0%, 50%, and 75% indicating low, moderate, and high heterogeneity, respectively [51].

Funnel plots were produced and examined for each of the main outcome measures (psychological distress, anxiety, depression, and trauma symptoms), to test for publication bias within included studies [52]. Egger's test of the intercept was used to measure the significance level of any potential publication bias identified [53]. To account for any potential publication bias, Duval and Tweedie's trim and fill procedure [54] was performed to compute corrected effect sizes based on estimating the number of missing studies to account for any asymmetry in the funnel plot.

### 2.9. Meta Regression Analyses

To explore the influence of methodological, participant, intervention, and delivery level differences between studies on outcomes at posttreatment, a number of univariate meta-regression analyses were conducted using Stata 17 [47]. Nine different moderators were included in the analyses: age (<39 years = 1; <40 years = 2), gender (<50% female dominated = 1; <50% male dominated = 2), geographic location of country (Asia = 1; Africa = 2; South America = 3), recruitment (Community = 1; Primary Care = 2; Both = 3), country income classification (Low-income country = 1; lower-middle income country = 2; upper-middle income country = 3), intervention type (PM+ = 1; CETA = 2; Other = 3), mean number of sessions (< = 6 sessions = 1; > 6 sessions = 2), mean session length (< = 60 min = 1; > 60 min = 2), intervention format (Group = 1; Individual = 2; Individual and Group = 3; App = 4), NSP training length (< = 7 days = 1; > 7 days = 2), and control type (ETAU = 1; W/*L* = 2; Other = 3).

### 2.10. Sensitivity Analysis

To test the effect of quality appraisal scores on effect sizes, a sensitivity analysis was performed by critically appraising the study's overall methodological quality based on the Cochrane “Risk of Bias tool” [45].

## 3. Results

### 3.1. Descriptive Characteristics of the Included Studies

**Figure 1:**
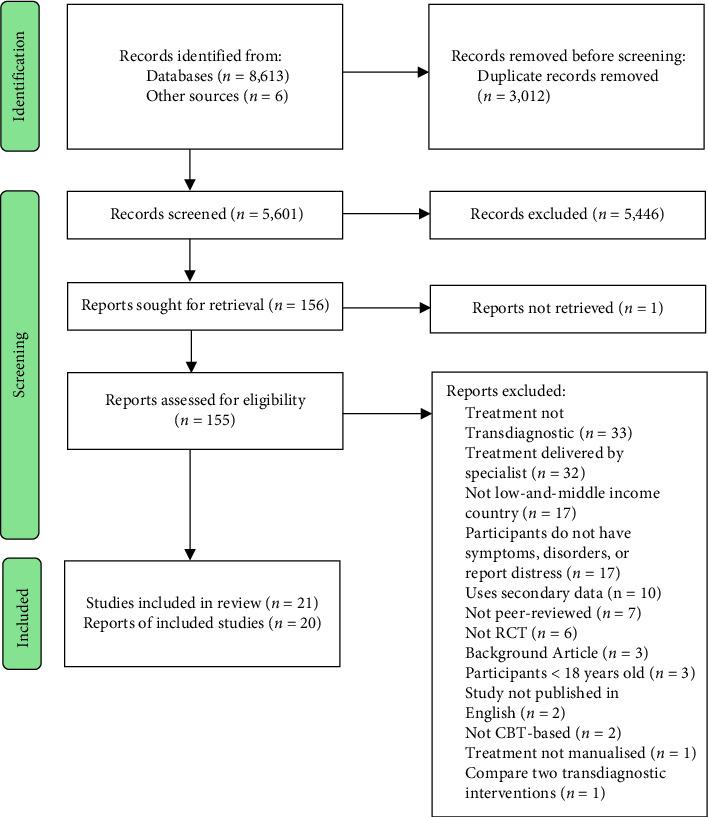
PRISMA diagram of included studies.

In total, we retrieved 8,613 articles (see Figure 1). Of these, 3012 were duplicates with a further 5,446 articles removed due to not meeting the inclusion criteria, leaving 156 articles for full-text screening. An additional 134 studies were excluded as they (a) did not report interventions delivered by an NSP (b) included non-transdiagnostic interventions, (c) were not completed in a LAMIC, (d) did not include participants reporting symptoms of CMDs or NPD, (e) used secondary data, (f) were not peer reviewed, (g) were not an RCT, (h) did not report data, (i) were not published in English, (j) were not CBT-based (k) were not accessible (l) the treatment was not manualized or (m) compared two transdiagnostic interventions. A total of 21 trials across 20 papers were included in the review, with one paper reporting two separate studies.

Studies were completed across a range of different LAMIC countries including Pakistan (*n* = 4), Türkiye (*n* = 3), Colombia (*n* = 2), Jordan (*n* = 2), Kenya (*n* = 2), and Nepal (*n* = 2), with one study each conducted in Iraq, Malaysia, Thailand, Uganda, Zambia, and Zimbabwe. The age of the participants ranged between 18 and 85 years old (Mean age = 38.1; SD = 4.93) with 81% of the overall sample of 5843 participants identifying as female.

For the psychological interventions, 52% (*n* = 11) of the studies used a face-to-face individual format and 38% (*n* = 8) used group sessions while two studies used a mixed individua/group format (10%). Versions of problem management plus (PM+) were used in 52% of studies (*n* = 11), CETA or adaptions of this were used in 24% of studies (*n* = 5), while the remaining 24% trials (*n* = 5) included a variety of transdiagnostic interventions (Culturally adapted CBT, Self-Help Plus, Friendship Bench, and CBT). The average number of sessions was 6.7 (SD = 2.6), with a mean overall length of 102 min (SD = 33.2 min; range: 37–180 min.). Mean length of training for NSPs was 8.9 days (SD = 3days; range: 4.5–20 days.). 72% of control arms were enhanced treatment as usual (*n* = 15), with 24% being a waiting list control (*n* = 5) and the remaining study consisting of an active comparison. Participants were recruited via the community or refugee camps in 66% of studies (*n* = 14), with 24% of studies recruiting participants via primary care clinics (*n* = 5) and 10% of studies using a mixture of primary care and community recruitment (*n* = 2). Only 10% of studies (*n* = 2) assessed the cost-effectiveness of their interventions, while 33% of studies assessed acceptability and feasibility (*n* = 7). All studies mentioned the number of participants lost to follow-up, while all but 24% (*n* = 5) of studies provided information on any adverse events. Adverse events related to the intervention were reported in only 10% (*n* = 2) of studies, with six incidents of suicidal ideation recorded in one study, while in the other study, one incident of attempted suicide and one case of hospitalization due to severe depression were reported. Full characteristics of all included studies can be found in Table 5 below.

### 3.2. Assessment of Risk of Bias

**Figure 2:**
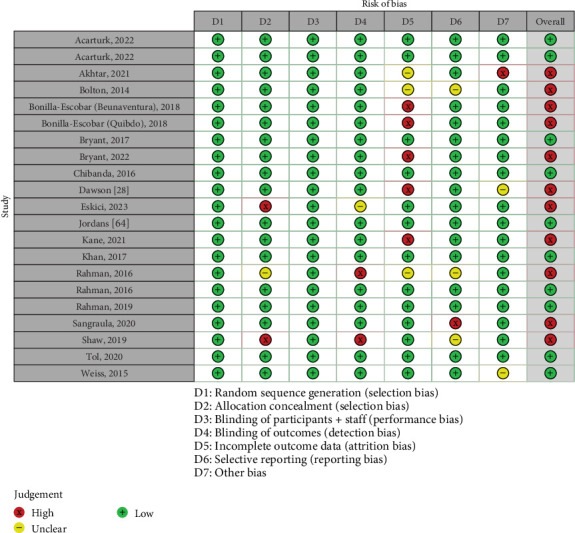
Risk of bias assessment for included studies.

The methodological appraisal exercise demonstrated that more than half of the included studies (*n* = 11; 52%) showed high risk of bias (see Figure 2 below).

### 3.3. Outcomes

#### 3.3.1. Psychological Distress at Posttreatment and Follow-Up

**Figure 3:**
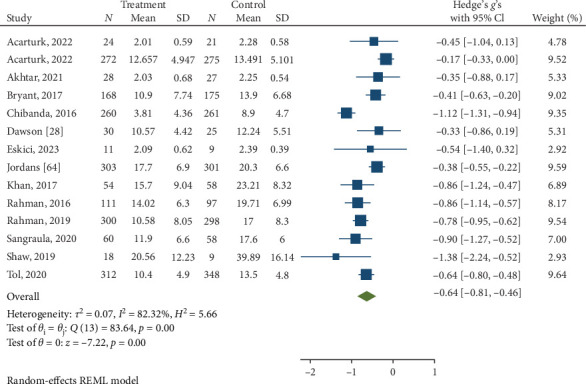
Reductions in NPD at posttreatment.

**Figure 4:**
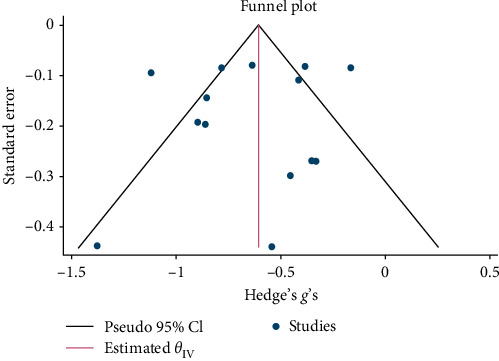
Funnel plot for effect sizes for reduction in NPD.

**Figure 5:**
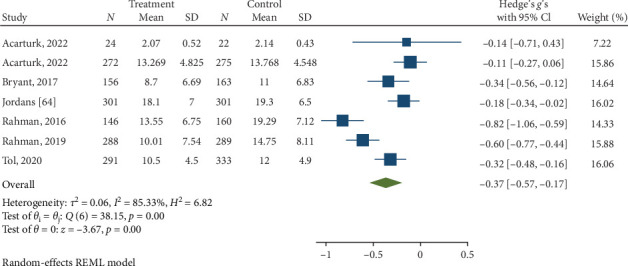
Reductions in NPD at follow-up.

Psychological interventions yielded a significant and moderately sized pooled effect (g = −0.64; 95% CI: −0.81 to −0.46; *p* = .01; Figure 3) [65] for reducing the severity of distress at posttreatment, based on 14 unique comparisons. However, heterogeneity was high, I^2^ = 82.32%. There was no indication of publication bias (*p*  > 0.05; for Funnel plots, see Figure 4), as assessed using Egger's test. At follow-up, spanning a period ranging between 13 and 26 weeks, with a mean of 14.6 weeks (SD = 4.5), psychological interventions demonstrated a small, pooled effect size (SMD = −0.37; 95% CI: −0.57 to −0.17; *p*=0.01; Figure 5) based on seven comparisons, with high heterogeneity, I^2^ = 85.33%.

#### 3.3.2. Anxiety Symptoms at Posttreatment and Follow-Up

**Figure 6:**
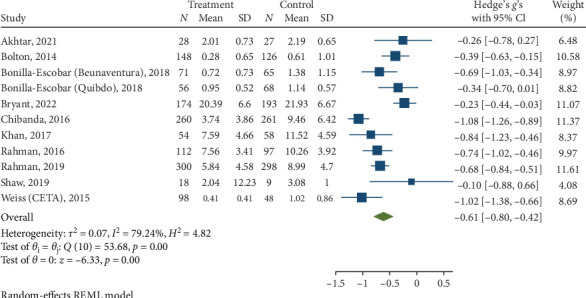
Reductions in anxiety at posttreatment.

**Figure 7:**
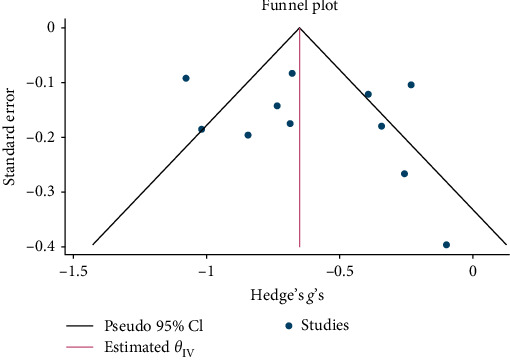
Funnel plot for effect sizes for reduction in anxiety.

**Figure 8:**
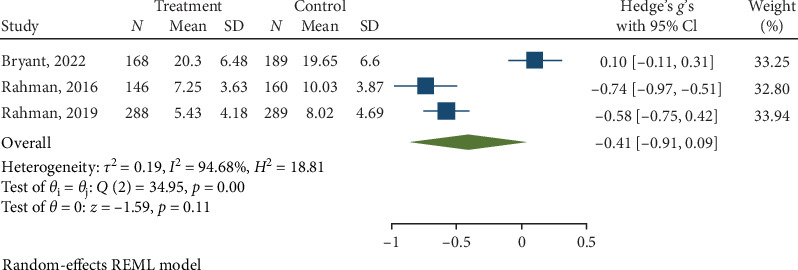
Reductions in anxiety at follow-up.

Psychological interventions yielded a significant and moderately sized pooled effect (g = −0.61; 95% CI: −0.80 to −0.42; *p*=0.01; Figure 6) in reducing severity of anxiety posttreatment, drawing from 11 distinct comparisons. However, there was notably high heterogeneity (I^2^ = 79.24%). No signs of publication bias were evident (*p*  > 0.05; Figure 7) as determined by Egger's test. During the follow-up period of 13 weeks, psychological interventions showed a small, pooled effect size (SMD = − 0.41; 95% CI: −0.91 to 0.09; *p*=0.01; Figure 8) based on three comparisons. Heterogeneity was high (I^2^ = 94.68%).

#### 3.3.3. Depression Symptoms at Posttreatment and Follow-Up

**Figure 9:**
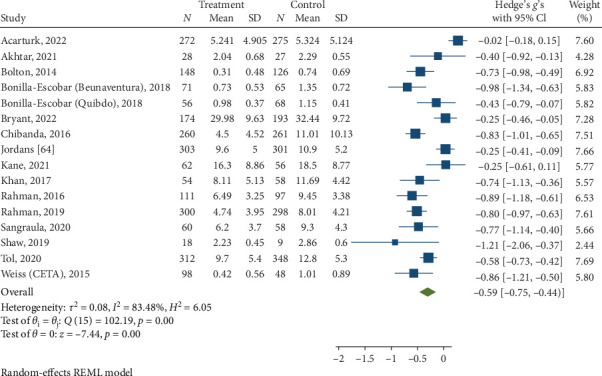
Reductions in depression at posttreatment.

**Figure 10:**
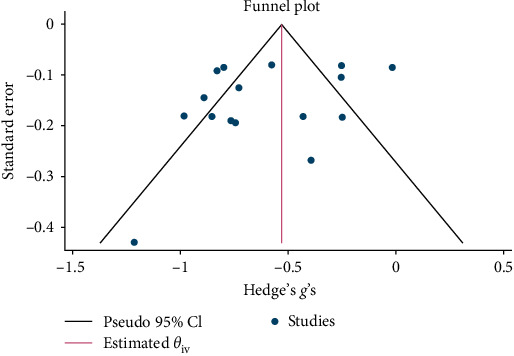
Funnel plot for effect sizes for reduction in depression.

**Figure 11:**
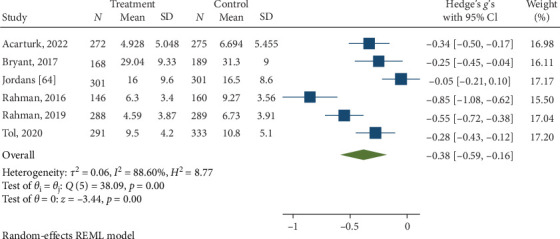
Reductions in depression at follow-up.

Psychological interventions produced a significant and moderately sized pooled effect (g = −0.59; 95% CI: −0.75 to −0.44; *p*=0.01; Figure 9) in reducing severity of depression posttreatment, drawing from 16 distinct comparisons. However, heterogeneity was high (I^2^ = 83.48%). No signs of publication bias were evident (*p*  > 0.05; Figure 10), as determined by Egger's test. During the follow-up period, ranging from 13 to 26 weeks, with a mean of 14.8 weeks (SD = 4.5), psychological interventions showed a significant and small-sized pooled effect (g = −0.38; 95% CI: −0.59 to −0.16; *p*=0.01; Figure 11) based on six comparisons, and heterogeneity remained high (I^2^ = 88.60%).

#### 3.3.4. PTSD/Trauma Symptoms at Posttreatment and Follow-Up

**Figure 12:**
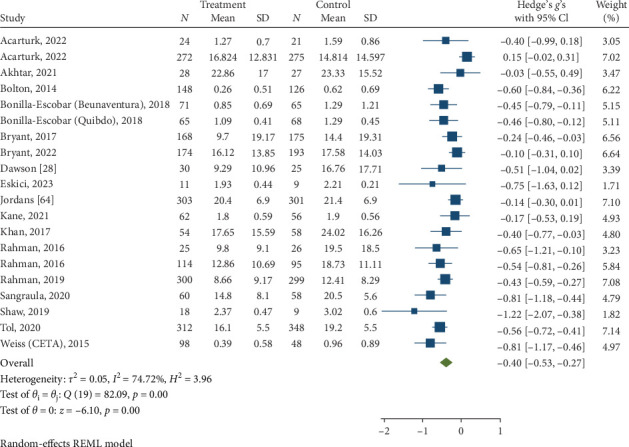
Reductions in trauma/post-traumatic stress disorder (PTSD) at posttreatment.

**Figure 13:**
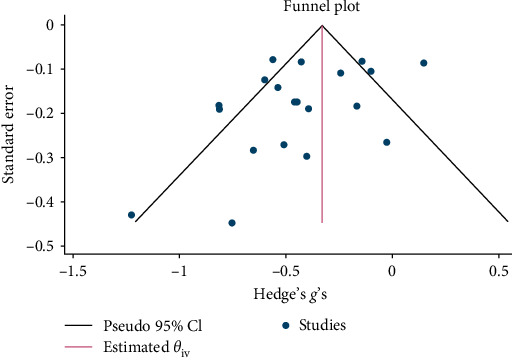
Funnel plot for effect sizes for reduction in trauma/PTSD.

**Figure 14:**
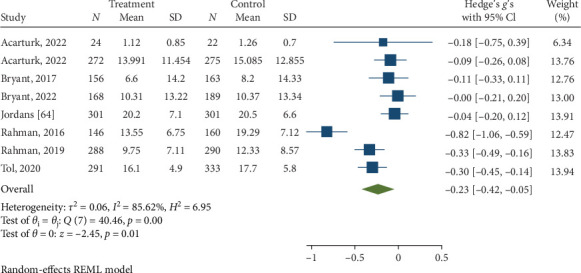
Reductions in trauma/PTSD at follow-up.

Psychological interventions produced a significant and small-sized pooled effect (g = −0.40; 95% CI: −0.53 to −0.27; *p* = .01; Figure 12) in reducing severity of PTSD/trauma symptoms posttreatment, drawing from 20 distinct comparisons. However, heterogeneity was very high (I^2^ = 74.72%). No signs of publication bias were evident (*p*  > 0.05; Figure 13), as determined by Egger's test. During the follow-up period, ranging from 13 to 26 weeks, with a mean of 14.4 weeks (SD = 4.1), psychological interventions showed a significant but small-sized pooled effect (g = −0.23; 95% CI: −0.42 to −0.05; *p*=0.01; Figure 14) based on eight comparisons, and heterogeneity remained relatively high (I^2^ = 85.62%).

### 3.4. Meta-Regression Analysis

Univariate meta-regression analyses examining the effects of the psychological interventions on the reduction of trauma/PTSD symptom at posttreatment demonstrated that studies using longer sessions contributed larger effect sizes (*b* = 0.37 (95% CI = 0.02, 0.73, *p*=0.04). This means that longer sessions were more beneficial in reducing distress compared to those interventions that were based on shorter sessions (e.g., 60 min or less).

Univariate meta-regression analyses for the effects of the psychological interventions on the reduction of anxiety symptoms at posttreatment demonstrated that higher risk studies (*b* = 0.50 95% CI = 0.26, 0.74), *p*=0.001) contributed larger effect sizes compared to lower risk studies, implying that studies with poorer quality were reported to be more effective than those with better quality.

All other meta-regression analyses assessing the impact of psychological interventions on the severity of anxiety, depression, and PTSD/trauma symptoms did not yield significant outcomes at posttreatment. This indicates that the examined characteristics did not have a discernible effect on the overall effectiveness of these psychological interventions in addressing these outcomes. However, these findings should be interpreted with caution due to the limited number of studies among the different comparison groups.

## 4. Discussion

### 4.1. Summary and Interpretation of Findings

We conducted a systematic review and meta-analysis to examine how effective transdiagnostic psychological interventions were in treating symptoms of CMDs and NPD in LAMICs. We focused exclusively on those interventions that were carried out by NSPs because of the scarcity of trained mental health professionals in delivering psychological interventions in these countries. We also focused on distress, anxiety, depression, and PTSD/trauma outcomes because these symptoms are routinely screened in LAMICs, and included those that both had a formal diagnosis and self-reported symptoms. Our findings demonstrated moderate reductions in distress, and symptoms of anxiety, depression, and PTSD/trauma, which were maintained at follow-ups spanning up to 26 weeks after the termination of the therapy. These findings are important because they demonstrate the effectiveness of transdiagnostic psychological interventions for symptoms of CMDs and NPD, even when these were delivered by NSPs.

Our meta-regression analyses demonstrated the superiority of longer therapeutic sessions, but only for trauma/PTSD symptoms. Studies with a higher risk of bias, compared to those with a lower risk of bias, contributed larger effect sizes for anxiety symptoms. These are interesting findings that deserve further exploration. However, we need to stress out that the number of studies focussing on anxiety was small (only 11 studies) and, as such, no firm conclusions can be drawn. The number of studies contributing to the trauma/PTSD outcomes was more substantial; we found an initial indication of the superiority of the longer sessions of trauma/PTSD reductions. These are interesting findings that also deserve further exploration from future studies.

Our analyses agree with previous reviews that have documented the effectiveness of various psychological treatments for reducing symptoms of CMDs in adults living in LAMIC [75, 76], including those delivered by NSPs [34, 77, 78]. We have also replicated the effects of transdiagnostic therapies in reducing symptoms of depression and anxiety, as previously shown in HIC [37, 79, 80]. It is noteworthy that the study of Newby et al. [37] focusing on treatments delivered by mental health professionals showed large effect sizes for anxiety and depression in uncontrolled studies. However, upon closer examination of controlled studies, the effect sizes remained high only for depression, while the effect size for reductions in anxiety measures was moderate. The risk of bias in most included studies was high.

Our review is in accord with the study by van Ginneken et al. [81], which not only focused on those with symptoms or a formal diagnosis of CMDs delivered by NSPs in adult populations residing in LAMICs but also targeted various mental health disorders including dementia and other severe conditions. These interventions were administered by a diverse range of health workers—both professionals and nonprofessionals—across populations of different age groups. They reported a moderate effect size for CMD treatments when delivered by lay health workers. Our review differs from these previous studies by focussing exclusively on transdiagnostic psychological treatments for symptoms of CMDs, namely distress, anxiety, depression, and PTSD/trauma, delivered by NSPs.

The current review provides insight into scalable interventions for LAMICs while identifying further gaps in knowledge. The high level of participants being recruited through the community rather than through formal health settings is unsurprising given the low level of access to mental health support, including within primary care, in LAMICs [82]. Only a third of included studies examined acceptability and feasibility. However, some of the included trials had been preceded by non-randomized feasibility studies that had established the acceptability of the interventions [83] or were themselves larger efficacy trials of some of the included feasibility trials [65, 69]. Despite this, there was a noticeable absence of cost-effectiveness measures included. This underlies one of the key challenges in how transdiagnostic interventions may be sustained within local settings following the completion of trials, given the barriers to accessing resources within LAMIC. Goloktionova and Mukerjee [84] reported that, despite a pilot demonstrating that PM + was successful in treating CMD within a conflict effected region of eastern Ukraine, there have been major difficulties in continuing in primary care health settings without the backing of government-level support. Little research has focused on the sustainability of transdiagnostic interventions delivered by NSPs on a long-term basis, particularly once support from NGOs, researchers, and associated grants has been reduced. It will be important that the sustainability and cost-effectiveness of task-shifting transdiagnostic interventions are considered, to examine to what extent the benefits of these approaches are sustained, and how they might be integrated within local health systems. The use of passive controls in all but one study means it is unclear if transdiagnostic interventions would remain as effective as single disorder interventions, as has been questioned in HIC populations [85]. However, the low level of formal diagnostic tools used in the included studies suggests that accurately diagnosing specific disorders continues to be major challenge within LAMICs [69, 86]. This may mean that transdiagnostic approaches that are not reliant on accurate diagnosis are preferable within poorly resourced health systems. While examining other promising low-cost interventions, such as digital self-help interventions [87, 88, 89] was beyond the focus of the current study, it will also be important to examine how these compare to transdiagnostic interventions in terms of efficacy and cost-effectiveness given the increasing access to internet in LAMICs [90].

### 4.2. Strengths and Weaknesses

This is the most comprehensive systematic review and meta-analysis, comprising 20 studies providing data on 21 unique trials focusing on the effectiveness of transdiagnostic psychological interventions for symptoms of CMDs and NPD delivered by NSPs in LAMICs. Our study uniquely focused on distress, anxiety, depression, and PTSD/trauma symptoms, making it the first review to comprehensively address a spectrum of common mental health difficulties. Furthermore, we employed formal tests to evaluate risk of bias and heterogeneity and implemented methods to address any potential publication bias. Last, the methodological assessment revealed a high risk of bias in most of the studies included.

Our review has six key limitations that should be discussed. First, the heterogeneity across the included studies was high across all four outcomes, namely distress, anxiety, depression, and PTSD/trauma symptoms. While this reflects the studies being conducted across a wide range of countries and settings, we employed random-effect models to account for both within-study and between-study variability. Second, the number of comparisons, although not small, was limited for most outcomes, particularly for distress and anxiety (14 and 11 unique comparisons per outcome respectively). Third, some of the included studies were based on a small or moderate number of participants. To account for this, we used Hedge's *g* as our preferred effect size index, which corrects for sample size biases. Fourth, the meta-regression analyses may have lacked sufficient power, requiring careful interpretation. Due to the limited number of comparisons, we did not perform meta-regression analyses for the follow-up outcomes. Fifth, all but one included study relied on self-report screening measures to assess level of symptomology in participants, meaning the review could not establish if most participants had or would have received a formal diagnosis of a CMD, and instead focused on symptoms of CMDs or NPD. While inclusion of formal clinician diagnoses is less common in trials completed in LAMIC given the lower level of access to mental health services, García-Escalera et al. [35] found that studies which did not report participants' diagnoses showed higher effect sizes than both studies which limited inclusion to those with a clinical diagnosis suggesting potential bias. It remains to be established if the same effect sizes would be found focusing on only those participants with a formal diagnosis of a CMD. Finally, while the transdiagnostic interventions included were shown to work within controlled research studies, it is unclear to what degree these same outcomes may be achieved within local care systems.

### 4.3. Implications for Clinicians and Policy Makers and Future Directions

Our findings have important clinical and research implications. Firstly, it is apparent that the delivery of such psychological interventions by NSPs is effective after minimal training, and their effectiveness is maintained at follow-up, establishing the role of NSPs as very important for task shifting, especially in countries that lack trained mental health professionals. Therefore, we recommend continuing task-shifting to NSPs in order to address the mental health treatment gap and for this to be expanded beyond research studies and trialed within local care systems. Particular focus should be given to examining the cost-effectiveness and long-term sustainability of these interventions, as this remains a gap in knowledge. Secondly, our findings confirm that manualized transdiagnostic approaches can be effective at treating a range of different symptoms of CMDs and NPD. While the treatment effect sizes suggest that approaches that target common aspects across conditions can be effective when delivered by NSPs, in most studies transdiagnostic interventions were compared to a non-active comparator, such as enhanced usual care. It will be important for future research to examine how these transdiagnostic approaches compare to disorder-specific interventions, as well as digital interventions, as research in HIC suggests there may be differences [85]. Thirdly, there was an initial indication that longer sessions may be more beneficial than shorter ones, particularly for reducing PTSD/trauma symptoms. Therefore, it is important to incorporate flexibility in treatment planning to allow for extended sessions, if necessary. Finally, the quality of the included studies was low, while most of the participants only self-reporting symptoms and lacked a formal diagnosis of a CMD. This highlights the need for more high-quality research to be conducted in this important area, given the need for psychological support in LAMICS and the number of people potentially benefitting from these approaches. Overall, our findings are important and unique because they advocate for policies promoting access to mental health interventions in LAMICs, with an emphasis on transdiagnostic approaches delivered by NSPs.

### 4.4. Conclusions

This review has shown that transdiagnostic psychological interventions delivered by NSPs are moderately effective at reducing symptoms of distress, anxiety, depression, and trauma in LAMICs. This effect, although reduced, was maintained for each outcome at follow-up. Overall, this review supports the continued task shifting of psychological interventions to NSPs in LAMICs because it was proven effective in regions lacking mental health professionals. These findings suggest that researchers should continue exploring the potential benefits of task-shifting in LAMICs. It also would be interesting for future research to compare task-shifting approaches with disorder-specific interventions. Last, although these findings support the effectiveness of longer sessions, further exploration across different mental health conditions is warranted.

## Figures and Tables

**Table 1:** Overview of different non-specialist providers.

Non-specialist providers identifiers used
Lay health workers, lady health workers, volunteer health workers, voluntary health workers, community health workers, community health distributors, community health surveyors, community health assistants, community health promoters, rural health auxiliaries, promotoras de salud, paraprofessionals auxiliary health staff, midwives, nurses, teachers, doctors, peers, refugees, religious, and traditional healers

**Table 2:** Overview of different transdiagnostic approaches as outlined by Murray et al. [27].

Name	Descriptor
Universally applied therapeutic principles	Encompass specific “schools” of psychotherapy, such as cognitive-behavioral [34] and psychodynamic therapy [35], which apply certain key techniques across a range of psychopathologies, using a “top down” approach.

Modular treatments	Also known as common elements approaches, these treatments target a range of disorders by allowing clinicians to assemble different therapeutic components within a treatment, to treat the individuals' presenting problems [32].

Shared- mechanism treatments	These interventions are used to address multiple disorders by targeting common mechanisms that underlie numerous disorders.

Principle-guided approaches	Treatments that apply and combine a range of core evidenced-based therapeutic principles, such as feeling calm, increasing motivation, and repairing thoughts, to address multiple different psychopathologies [33].

**Table 3:** PRISMA checklist.

Section and Topic	Item #	Checklist item	Location where item is reported
Title			—
Title	1	Identify the report as a systematic review.	1
Abstract			—
Abstract	2	See the PRISMA 2020 for Abstracts checklist.	2
Introduction			—
Rationale	3	Describe the rationale for the review in the context of existing knowledge.	3−7
Objectives	4	Provide an explicit statement of the objective(s) or question(s) the review addresses.	7
Methods			—
Eligibility criteria	5	Specify the inclusion and exclusion criteria for the review and how studies were grouped for the syntheses.	7−9
Information sources	6	Specify all databases, registers, websites, organizations, reference lists, and other sources searched or consulted to identify studies. Specify the date when each source was last searched or consulted.	9
Search strategy	7	Present the full search strategies for all databases, registers, and websites, including any filters and limits used.	9−10
Selection process	8	Specify the methods used to decide whether a study met the inclusion criteria of the review, including how many reviewers screened each record and each report retrieved, whether they worked independently, and if applicable, details of automation tools used in the process.	10
Data collection process	9	Specify the methods used to collect data from reports, including how many reviewers collected data from each report, whether they worked independently, any processes for obtaining or confirming data from study investigators, and if applicable, details of automation tools used in the process.	10−11
Data items	10a	List and define all outcomes for which data were sought. Specify whether all results that were compatible with each outcome domain in each study were sought (e.g. for all measures, time points, analyses), and if not, the methods used to decide which results to collect.	10
	10b	List and define all other variables for which data were sought (e.g. participant and intervention characteristics, funding sources). Describe any assumptions made about any missing or unclear information.	10−11
Study risk of bias assessment	11	Specify the methods used to assess risk of bias in the included studies, including details of the tool(s) used, how many reviewers assessed each study and whether they worked independently, and if applicable, details of automation tools used in the process.	11
Effect measures	12	Specify for each outcome the effect measure(s) (e.g. risk ratio, mean difference) used in the synthesis or presentation of results.	11−12
Synthesis methods	13a	Describe the processes used to decide which studies were eligible for each synthesis (e.g. tabulating the study intervention characteristics and comparing against the planned groups for each synthesis (item #5)).	10−12
	13b	Describe any methods required to prepare the data for presentation or synthesis, such as handling of missing summary statistics, or data conversions.	11−12
	13c	Describe any methods used to tabulate or visually display results of individual studies and syntheses.	12
	13d	Describe any methods used to synthesize results and provide a rationale for the choice(s). If meta-analysis was performed, describe the model(s), method(s) to identify the presence and extent of statistical heterogeneity, and software package(s) used.	11−12
	13e	Describe any methods used to explore possible causes of heterogeneity among study results (e.g. subgroup analysis, meta-regression).	12−13
	13f	Describe any sensitivity analyses conducted to assess robustness of the synthesized results.	13
Reporting bias assessment	14	Describe any methods used to assess risk of bias due to missing results in a synthesis (arising from reporting biases).	11
Certainty assessment	15	Describe any methods used to assess certainty (or confidence) in the body of evidence for an outcome.	11
Results			—
Study selection	16a	Describe the results of the search and selection process, from the number of records identified in the search to the number of studies included in the review, ideally using a flow diagram.	13−15
	16b	Cite studies that might appear to meet the inclusion criteria, but which were excluded, and explain why they were excluded.	13
Study characteristics	17	Cite each included study and present its characteristics.	16−30
Risk of bias in studies	18	Present assessments of risk of bias for each included study.	31
Results of individual studies	19	For all outcomes, present, for each study: (a) summary statistics for each group (where appropriate) and (b) an effect estimate and its precision (e.g. confidence/credible interval), ideally using structured tables or plots.	32−39
Results of syntheses	20a	For each synthesis, briefly summarize the characteristics and risk of bias among contributing studies.	39−40
	20b	Present results of all statistical syntheses conducted. If meta-analysis was done, present for each the summary estimate and its precision (e.g. confidence/credible interval) and measures of statistical heterogeneity. If comparing groups, describe the direction of the effect.	32−39
	20c	Present results of all investigations of possible causes of heterogeneity among study results.	39−40
	20d	Present results of all sensitivity analyses conducted to assess the robustness of the synthesized results.	39−40
Reporting biases	21	Present assessments of risk of bias due to missing results (arising from reporting biases) for each synthesis assessed.	31
Certainty of evidence	22	Present assessments of certainty (or confidence) in the body of evidence for each outcome assessed.	32−39
Discussion			—
Discussion	23a	Provide a general interpretation of the results in the context of other evidence.	40−43
	23b	Discuss any limitations of the evidence included in the review.	43−44
	23c	Discuss any limitations of the review processes used.	43−44
	23d	Discuss implications of the results for practice, policy, and future research.	44−45
Other Information			—
Registration and protocol	24a	Provide registration information for the review, including register name and registration number, or state that the review was not registered.	7
	24b	Indicate where the review protocol can be accessed, or state that a protocol was not prepared.	7
	24c	Describe and explain any amendments to information provided at registration or in the protocol.	7
Support	25	Describe sources of financial or non-financial support for the review, and the role of the funders or sponsors in the review.	46
Competing interests	26	Declare any competing interests of review authors.	46
Availability of data, code, and other materials	27	Report which of the following are publicly available and where they can be found: template data collection forms; data extracted from included studies; data used for all analyses; analytic code; any other materials used in the review.	46

**Table 4:** Search terms.

#	Query
1	(Open trial or RCT or randomized controlled or randomized controlled or randomized controlled study or randomized controlled study or randomized controlled trial or randomized controlled trials or randomized controlled trial or randomized controlled trials or random controlled trial or effectiveness study or clinical trial or controlled trial or controlled study or randomized study or randomized study or random study or randomized cross-over trial or randomized cross-over trial or randomized cross-over study or randomized cross-over study or cross-over trial or cluster randomized trial or cluster-randomization or cluster-randomization or cluster randomization or cluster randomization or cluster randomized trial or quasi-experimental study or controlled clinical trial or multicenter study or trial or double blind or double-blind or double-blind procedure or randomization or Randomization or single blind or single-blind or single blind procedure or single-blind procedure).mp. (m*p* = title, abstract, heading word, drug trade name, original title, device manufacturer, drug manufacturer, device trade name, keyword heading word, floating subheading word, candidate term word)
2	(((((Psychotherapy or therapy or cognitive therapy or counseling or counseling or Psychosocial Intervention or Psychotherapy, Brief or Psychotherapy, Group or Psychotherapy, Multiple or Psychological treatment or psychological intervention or psychodynamic or psychodynamic therapy or Psychotherapy, Psychodynamic or Psychodynamic psychotherapy or psychoanalytic or Psychotherapy, Rational-Emotive or psychoanalytic psychotherapy or cognitive therapy or therapy or treatment outcome or exposure therapy or cognitive behavioral therapy or cognitive behavior therapy or cognitive behavior therapy or cognitive behavioral therapy or behavior therapy or behavior therapy or acceptance) and commitment therapy) or Acceptance) and Commitment) or Dialectical Behavior Therapy or mindfulness based cognitive therapy or mindfulness-based cognitive therapy or compassion focused therapy or compassion-focused therapy or mindfulness treatments or mindfulness or mindfulness based stress reduction or dialectical behavior therapy or meta-cognitive therapy or metacognitive therapy or behavioral activation or psychotherapy outcome or anxiety treatment or depression treatment or unified protocol or transdiagnostic therapy or transdiagnostic or trans-diagnostic or Interpersonal Psychotherapy or interpersonal therapy or inter-personal therapy or inter personal therapy or common elements treatment approach or common elements or behavioral activation or behavioral activation or psychological treatment or psychological intervention or treatment or Intervention or Problem Management Plus or PM + or Problem Management or Broad-spectrum or broad spectrum or mixed diagnosis or mixed-diagnosis or transdiagnostic cognitive behavioral therapy or transdiagnostic cognitive behavioral therapy or transdiagnostic cognitive behavior therapy or transdiagnostic cognitive behavior therapy or T-CBT or transdiagnostic CBT or CBT or IPT or DBT or CETA or modular treatment or modular treatments or shared mechanism or shared mechanisms or shared mechanisms treatment or common elements treatment).mp. (m*p* = title, abstract, heading word, drug trade name, original title, device manufacturer, drug manufacturer, device trade name, keyword heading word, floating subheading word, candidate term word)
3	((((((Depression and anxiety) or Depress*⁣*^*∗*^ or mixed anxiety) and depression) or MADD or MDD or anxious depression or mixed anxiety or seasonal affective disorder or depression or dysthymic disorder or depressive disorder or depressive disorders or depression disorder or depression disorders major depressive disorder or major depression or minor depression or mood disorder or MDD or Depressive Disorder, Treatment-Resistant or dysthymia or dysthymia or Dysthymic Disorder or Depressive Disorder, Major or anxiety or anxiety disorder or anxiety disorder NOS or generalized anxiety disorder or GAD or trait worry or generalized anxiety or worry or stress or anxiety disorders or Anxiety, Separation or separation anxiety or combat disorders or mood disorder or Affective Disorder*⁣*^*∗*^ or internalizing or internalizing or internalizing disorder or internalizing disorder or emotional disorder or fear or emotional or distress or emotional distress or low mood or severe health anxiety or health anxiety disorder or illness anxiety disorder or hypochondriasis or hypochondriasis or obsessive behavior or hoarding or Hoarding Disorder or obsessive-compulsive disorder or obsessive-compulsive disorder or OCD or phobic or phobia or phobic disorders or Phobia, Social or Acrophobia or Agoraphobia or Claustrophobia or Ophidiophobia or post-traumatic or postpartum stress disorder or stress reaction or PTSD or post-traumatic stress or post-traumatic stress or acute stress disorder or acute stress or anxiety symptoms or Post-traumatic Stress Disorder or Complex PTSD or DESNOS or traumatic stress or psychological trauma*⁣*^*∗*^ or social phobia or social anxiety disorder or social anxiety or performance anxiety or fear of negative evaluation or Panic or panic disorder or panic attacks or panic disorder with agoraphobia or agoraphobia or comorbidity or comorbidity or co-morbid or transdiagnostic or trans-diagnostic or mixed anxiety) and depression) or anxious depression or mixed anxiety disorders or stressor or mixed diagnosis or mixed-diagnosis or Comorbid*⁣*^*∗*^ or Psychological distress or Emotional disorders or Emotional disorder or Common mental health or Common mental health condition or Common mental health conditions or Common mental disorders or Common mental disorder or Common mental health disorder or Common mental health disorders or CMD or Stress disorders or Neurotic Disorders).mp. (m*p* = title, abstract, heading word, drug trade name, original title, device manufacturer, drug manufacturer, device trade name, keyword heading word, floating subheading word, candidate term word)
4	(Task shifting or task sharing or task-shifting or task-sharing or local providers or NSP or non specialists or non specialist or non-specialist or local-provider or local provider or community healthcare worker or Task Switching or Task-Switching or community health workers or community health workers or community health worker or facilitator or facilitators or community based organization or health workforce or manpower or health manpower or lay or counselor or counselors or counselor or counseling or counseling or counselors or counselors or lay counselor or lay counselor or lay health worker or non-licensed or nonlicensed or non-licenced or nonlicenced or nonprofessional or nonprofessional or nonspecialist or non-specialists or nonspecialist or nonspecialists or patient care team or patient care teams or patient navigation or patient navigation or navigator or patient navigator or peer or peer-coach or peer-counselor or peer-counselor or peer-facilitator or promotor or promotors or promotora or promotoras or promotores or shared care or healer or healers or traditional healer or CHW or Cultural adaptation or Culture specific or Culture adaptation or Adaptation or adapted or Culturally adapted treatment or Culturally adapted therapy or Culturally Sensitive or culturally adapted or culturally relevant or cultural modification or culturally tailored or transculture or transcultural or culture sensitive or trans-culture or trans-cultural or culture or cultural or local or local adaptation).mp. (m*p* = title, abstract, heading word, drug trade name, original title, device manufacturer, drug manufacturer, device trade name, keyword heading word, floating subheading word, candidate term word)
5	(Afghanistan or Albania or Algeria or Angola or Antigua or Barbuda or Argentina or Armenia or Armenian or Aruba or Azerbaijan or Bahrain or Bangladesh or Barbados or Benin or Byelarus or Byelorussian or Belarus or Belorussian or Belorussia or Belize or Bhutan or Bolivia or Bosnia or Herzegovina or Hercegovina or Botswana or Brasil or Brazil or Bulgaria or Burkina or Faso or Fasso or Volta or Burundi or Urundi or Cambodia or Khmer or Kampuchea or Cameroon or Cameroons or Cameron or Camerons or Cape Verde or African or Chad or Chile or China or Colombia or Comoros or Comoro or Comores or Mayotte or Congo or Zaire or Rica or d Ivoire or Ivory or Cuba or Djibouti or Somaliland or Dominica or Dominican or Timor or Timur or Leste or Ecuador or Egypt or Arab or Salvador or Eritrea or Ethiopia or Fiji or Gabon or Gabonese or Gambia or Gaza or Georgia or Georgian or Ghana or Grenada or Guatemala or Guinea or Guam or Guiana or Guyana or Haiti or Honduras or Hungary or India or Maldives or Indonesia or Iran or Iraq or Jamaica or Jordan or Kazakhstan or Kazakh or Kenya or Kiribati or Korea or Kosovo or Kyrgyzstan or Kirghizia or Kyrgyz or Kirghiz or Kirgizstan or Lao or Laos or Lebanon or Lesotho or Basutoland or Liberia or Libya or Macedonia or Madagascar or Malagasy or Malaysia or Malaya or Malay or Sabah or Sarawak or Malawi or Nyasaland or Mali or Marshall or Mauritania or Mauritius or Agalega or Mexico or Micronesia or Moldova or Moldovian or Mongolia or Montenegro or Morocco or Ifni or Mozambique or Myanmar or Myanma or Burma or Namibia or Nepal or Antilles or Caledonia or Nicaragua or Niger or Nigeria or Mariana or Oman or Muscat or Pakistan or Palau or Palestine or Palestinian or Panama or Paraguay or Peru or Philippines or Philipines or Phillipines or Phillippines or Filipino or Puerto Rico or Romania or Rumania or Roumania or Russia or Russian or Rwanda or Ruanda or Kitts or Nevis or Lucia or Vincent or Grenadines or Samoa or Samoan or Navigator or Sao Tome or Saudi Arabia or Senegal or Serbia or Montenegro or Seychelles or Sierra or Leone or Sri or Lanka or Ceylon or Solomon or Somalia or Africa or Sudan or Suriname or Surinam or Swaziland or Syria or Tajikistan or Tadzhikistan or Tadjikistan or Tadzhik or Tanzania or Thailand or Togo or Togolese or Tonga or Trinidad or Tobago or Tunisia or Turkey or Turkmenistan or Turkmen or Uganda or Ukraine or Uruguay or USSR or Soviet or Uzbekistan or Uzbek or Vanuatu or Hebrides or Venezuela or Vietnam or Viet Nam or West-Bank or Yemen or Yugoslavia or Zambia or Zimbabwe or Rhodesia or Polynesia or Hong Kong or Israel or Macao or Macau or Qatar or Singapore or Emirates or Afghan or Albanian or Algerian or Angolan or Antiguan or Barbadian or Argentinean or Armenian or Aruban or Azerbaijani or Aziri or Bahraini or Bangladeshi or Beninese or Belarussian or Belizean or Bhutanese or Bolivian or Bosnian or Herzegovinian or Batswana or Brazilian or Bulgarian or Burkinabe or Burundian or Cambodian or Khmer or Cameroonian or Cape Verdian or African or Chadian or Chilean or Chinese or Colombian or Comoran or Mahorais or Congolese or Ivoirian or Cuban or Djibouti or Somali or somalian or Dominican or Atoni or Ecuadorian or Egyptian or Arab or Salvadorian or Eritrean or Ethiopian or Fijians or Gabonese or Gambian or Georgian or Ghanaian or Grenadian or Guatemalan or Guinean or Guamanian or chamorro or Guyanese or Haitian or Honduran or Hungarian or Indian or Maldivians or Indonesian or Iranian or Iraqi or Jamaican or Jordanian or Kazakh or Kenyan or Kiribati or Korean or Kosovar or Kyrgyzstani or Kyrgyz or Laotian or Latino or Lebanese or Basotho or Liberian or Libyan or Macedonian or Malagasy or Malaysian or Malay or Malawian or Malian or Marshallese or Mauritanian or Mauritian or Mexican or Micronesian or Moldovan or Mongolian or Montenegrin or Moroccan or Mozambican or Myanmarese or Burmese or Namibian or Nepalese or Antilles or Caledonians or Nicaraguan or Nigerian or Omani or Pakistani or Palauan or Palestinian or Panamanians or Paraguayan or Peruvian or Filipino or Philippino or Puerto Rican or Romanian or Russian or Rwandan or Lucian or Samoan or Saudi or Arabian or Senegalese or Serbian or Seychellois or Sierra Leonean or Sri Lankan or Solomon or African or Sudanese or Swazi or Syrian or Tajiks or Tanzanian or Thai or Togolese or Tonga or Trinidadian or Tobagonian or Tunisian or Turkish or Turkmen or Ugandan or Ukrainian or Uruguayan or Uzbek or Vanuatu or Venezuelan or Vietnamese or Yemeni or Yugoslavian or Zambian or Zimbabwean or Rhodesian or Polynesian or Israeli or Macanese or Qatari or Singaporean or Emirati).ab. or (Afghanistan or Albania or Algeria or Angola or Antigua or Barbuda or Argentina or Armenia or Armenian or Aruba or Azerbaijan or Bahrain or Bangladesh or Barbados or Benin or Byelarus or Byelorussian or Belarus or Belorussian or Belorussia or Belize or Bhutan or Bolivia or Bosnia or Herzegovina or Hercegovina or Botswana or Brasil or Brazil or Bulgaria or Burkina or Faso or Fasso or Volta or Burundi or Urundi or Cambodia or Khmer or Kampuchea or Cameroon or Cameroons or Cameron or Camerons or Cape Verde or African or Chad or Chile or China or Colombia or Comoros or Comoro or Comores or Mayotte or Congo or Zaire or Rica or d Ivoire or Ivory or Cuba or Djibouti or Somaliland or Dominica or Dominican or Timor or Timur or Leste or Ecuador or Egypt or Arab or Salvador or Eritrea or Ethiopia or Fiji or Gabon or Gabonese or Gambia or Gaza or Georgia or Georgian or Ghana or Grenada or Guatemala or Guinea or Guam or Guiana or Guyana or Haiti or Honduras or Hungary or India or Maldives or Indonesia or Iran or Iraq or Jamaica or Jordan or Kazakhstan or Kazakh or Kenya or Kiribati or Korea or Kosovo or Kyrgyzstan or Kirghizia or Kyrgyz or Kirghiz or Kirgizstan or Lao or Laos or Lebanon or Lesotho or Basutoland or Liberia or Libya or Macedonia or Madagascar or Malagasy or Malaysia or Malaya or Malay or Sabah or Sarawak or Malawi or Nyasaland or Mali or Marshall or Mauritania or Mauritius or Agalega or Mexico or Micronesia or Moldova or Moldovian or Mongolia or Montenegro or Morocco or Ifni or Mozambique or Myanmar or Myanma or Burma or Namibia or Nepal or Antilles or Caledonia or Nicaragua or Niger or Nigeria or Mariana or Oman or Muscat or Pakistan or Palau or Palestine or Palestinian or Panama or Paraguay or Peru or Philippines or Philipines or Phillipines or Phillippines or Filipino or Puerto Rico or Romania or Rumania or Roumania or Russia or Russian or Rwanda or Ruanda or Kitts or Nevis or Lucia or Vincent or Grenadines or Samoa or Samoan or Navigator or Sao Tome or Saudi Arabia or Senegal or Serbia or Montenegro or Seychelles or Sierra or Leone or Sri or Lanka or Ceylon or Solomon or Somalia or Africa or Sudan or Suriname or Surinam or Swaziland or Syria or Tajikistan or Tadzhikistan or Tadjikistan or Tadzhik or Tanzania or Thailand or Togo or Togolese or Tonga or Trinidad or Tobago or Tunisia or Turkey or Turkmenistan or Turkmen or Uganda or Ukraine or Uruguay or USSR or Soviet or Uzbekistan or Uzbek or Vanuatu or Hebrides or Venezuela or Vietnam or Viet Nam or West-Bank or Yemen or Yugoslavia or Zambia or Zimbabwe or Rhodesia or Polynesia or Hong Kong or Israel or Macao or Macau or Qatar or Singapore or Emirates or Afghan or Albanian or Algerian or Angolan or Antiguan or Barbadian or Argentinean or Armenian or Aruban or Azerbaijani or Aziri or Bahraini or Bangladeshi or Beninese or Belarussian or Belizean or Bhutanese or Bolivian or Bosnian or Herzegovinian or Batswana or Brazilian or Bulgarian or Burkinabe or Burundian or Cambodian or Khmer or Cameroonian or Cape Verdian or African or Chadian or Chilean or Chinese or Colombian or Comoran or Mahorais or Congolese or Ivoirian or Cuban or Djibouti or Somali or somalian or Dominican or Atoni or Ecuadorian or Egyptian or Arab or Salvadorian or Eritrean or Ethiopian or Fijians or Gabonese or Gambian or Georgian or Ghanaian or Grenadian or Guatemalan or Guinean or Guamanian or chamorro or Guyanese or Haitian or Honduran or Hungarian or Indian or Maldivians or Indonesian or Iranian or Iraqi or Jamaican or Jordanian or Kazakh or Kenyan or Kiribati or Korean or Kosovar or Kyrgyzstani or Kyrgyz or Laotian or Latino or Lebanese or Basotho or Liberian or Libyan or Macedonian or Malagasy or Malaysian or Malay or Malawian or Malian or Marshallese or Mauritanian or Mauritian or Mexican or Micronesian or Moldovan or Mongolian or Montenegrin or Moroccan or Mozambican or Myanmarese or Burmese or Namibian or Nepalese or Antilles or Caledonians or Nicaraguan or Nigerian or Omani or Pakistani or Palauan or Palestinian or Panamanians or Paraguayan or Peruvian or Filipino or Philippino or Puerto Rican or Romanian or Russian or Rwandan or Lucian or Samoan or Saudi or Arabian or Senegalese or Serbian or Seychellois or Sierra Leonean or Sri Lankan or Solomon or African or Sudanese or Swazi or Syrian or Tajiks or Tanzanian or Thai or Togolese or Tonga or Trinidadian or Tobagonian or Tunisian or Turkish or Turkmen or Ugandan or Ukrainian or Uruguayan or Uzbek or Vanuatu or Venezuelan or Vietnamese or Yemeni or Yugoslavian or Zambian or Zimbabwean or Rhodesian or Polynesian or Israeli or Macanese or Qatari or Singaporean or Emirati).ti. or (Developing Countries or developing countr*⁣*^*∗*^ or developing nation*⁣*^*∗*^ or developing population*⁣*^*∗*^ or developing econom*⁣*^*∗*^ or undeveloped countr*⁣*^*∗*^ or undeveloped nation*⁣*^*∗*^ or undeveloped economy or least developed countr*⁣*^*∗*^ or least developed economies or less-developed countr*⁣*^*∗*^ or less-developed nation*⁣*^*∗*^ or less-developed econom*⁣*^*∗*^ or lesser developed nation*⁣*^*∗*^ or under-developed countr*⁣*^*∗*^ or under-developed nation*⁣*^*∗*^ or underdeveloped countr*⁣*^*∗*^ or underdeveloped nation*⁣*^*∗*^ or underdeveloped population*⁣*^*∗*^ or underdeveloped econom*⁣*^*∗*^ or low income countr*⁣*^*∗*^ or middle income countr*⁣*^*∗*^ or low income nation*⁣*^*∗*^ or middle income nation*⁣*^*∗*^ or low income population*⁣*^*∗*^ or middle income population*⁣*^*∗*^ or low income econom*⁣*^*∗*^ or middle income econom*⁣*^*∗*^ or lower income countr*⁣*^*∗*^ or lower income nation*⁣*^*∗*^ or lower income population*⁣*^*∗*^ or lower income economies or low resource countr*⁣*^*∗*^ or lower resource countr*⁣*^*∗*^ or low resource nation*⁣*^*∗*^ or low resource population*⁣*^*∗*^ or underserved countr*⁣*^*∗*^ or underserved population*⁣*^*∗*^ or under-served population or under-served populations or deprived countries or deprived population or deprived populations or transitional nations or transitional econom*⁣*^*∗*^ or transition countr*⁣*^*∗*^ or transition nation*⁣*^*∗*^ or transition econom*⁣*^*∗*^ or lower resource setting*⁣*^*∗*^ or middle resource setting*⁣*^*∗*^ or Third World*⁣*^*∗*^ or south east asia*⁣*^*∗*^ or middle east*⁣*^*∗*^ or Low-and middle income country or LAMIC or developing or LMIC or LAMI or LMICS or Developing Country).af.
6	Limit 1 and 2 and 3 and 4 and 5 to (human and English language)
7	1 and 2 and 3 and 4 and 5

*Source*: Page et al. [39] The PRISMA 2020 statement: an updated guideline for reporting systematic reviews. BMJ 2021;372:n71. doi: 10.1136/bmj.n71 For more information, visit: http://www.prisma-statement.org/.

**Table 5:** Characteristics of included studies.

Author, year, and country	Randomisation; recruitment method	Age: mean, (SD), range (%) female/male	Total sample size; difficulties reported	Inclusion	Intervention control	Intervention format	Outcome measures	Deliverers of intervention (highest education level) duration of training
Acarturk et al. (2022) [55]	Individual	38.02, (10.9), not reported (NR)	46	(a) Aged ≥18 years old (b) a Syrian with a temporary protection status granted by the government (c) an Arabic speaker, (d) >15 on K10 and (e) >16 on the WHODAS 2.0	Group PM+	Group	Primary = HSCL-25	Peer refugees with at least 12 years of education
Turkey	Community	67.40% female	Psychological distress		Enhanced care as usual (ECAU)	5 weekly sessions of 120 min	Secondary = PCL-5, PSYCHLOPS, CSRI, PMLDC	8-day training, followed by weekly local group supervision by certified PM+ trainers.

Acarturk et al. (2022) [56]	Individual	31.5, (9), NR	642	(a) Aged ≥18 years old (b) able to speak and understand Arabic; (c) under temporary protection according to Law on Foreigners and International Protection; (d) ≥3 on GHQ-12 (e) consented to take part	Self-help plus	Group	Primary = MINI	Peer non-specialist facilitators with a refugee or migrant background
Turkey	Community	62.9% female	Psychological distress		ECAU	Five 2-hr sessions	Secondary = GHQ-12, PCL-5, PHQ-9, PSYCHLOPS, WHODAS 2.0, WHO-5, HTQ, PMLD	5 days of training

Akhtar et al. (2021) [57]	Individual	43, (7.2), NR	64	(a) Syrian refugee, (b) aged ≥18 years old, (c) had a child or dependent living in the household aged 10–16 years, and (d) scored ≥16 on the K10, and ≥17 on the WHODAS 2.0	Group PM+	Group	Primary = HSCL-25	Facilitators (non-specialists with a bachelor's degree in psychology or a field related to health)
Jordan	Community	70% female	Distress		Enhanced treatment-as-usual (ETAU)	5 weekly sessions of 120 min	Secondary = PCL-5, PSYCHLOPS, PG-13; PQ-B	8 days of training, followed by two supervised practice cycles

Bolton et al. (2014) [58]	Individual	35.45, (12.08), 18–85	347	Burmese individuals aged ≥18 years who (a) witnessed or experienced a traumatic event and (b) moderate to severe depression and/or PTSS based on HSCL-25 and the HTQ.	CETA	Individual	Primary = HSCL-25 and HTQ	Counselors (teachers, health workers, doctor, mental health counselor, former political prisoner, no information regarding specific level of education)
Thailand	Community	62.6% female	Depression and/or post-traumatic stress		Waiting list	Weekly 1-hr sessions (average 10 sessions)	Secondary = aggression questionnaire; AUDIT	10 days of in-person training followed by practice groups

Bonilla-Escobar et al. (2018; Beunaventura)[59]	Individual	41.1, (14.5), NR	180	(a) Aged >18 years (b) identifying as Afro-Colombian, (c) reporting having experienced at least one violent traumatic experience, (d) having a TMHS score greater than 0.77, and (e) having any reduced functionality in routine activities.	CETA	Individual	Primary = TMHS	Lay psychosocial community workers (LPCW; at least 5 years of post-primary education)
Colombia	Community	88.8% women	Post-traumatic stress disorder (PTSD)/ depression/ anxiety		Control group	12–14 weekly, 1.5-hr sessions	Secondary = HSCL-25, HTQ, PCL-C	10-day training by CETA experts, followed by weekly practice groups and then personalized supervision meetings

Bonilla-Escobar et al. (2018; Quibdo)[59]	Individual	45, (18.9), NR	166	(a) Aged ≥18 years (b) identifying as Afro-Colombian, (c) reporting having experienced at least one violent traumatic experience, (d) having a TMHS score greater than 0.77, and (e) having any reduced functionality in routine activities.	CETA	Individual	Primary = TMHS	LPCW (at least 5 years of post-primary education)
Colombia	Community	71.4% female	PTSD/ depression/ anxiety		Control group	12–14 weekly, 1.5-hr sessions	Secondary = HSCL-25, HTQ, PCL-C	10-day training by CETA experts, followed by weekly practice groups and then personalized supervision meetings

Bryant et al. (2017) [60]	Individual	35.55, (13.4), NR	421	A history of GBV, score ≥3 on the GHQ-12, and a score ≥17 on WHODAS 2.0	PM+	Individual	Primary = GHQ-12	CHWs (10 years' school education and did not have prior training or experience in mental healthcare.)
Kenya	Community	100% female	Psychological distress		Enhanced usual care (EUC)	5 weekly 90-minindividual sessions	Secondary = PCL-5, PSYCHLOPS, & WHODAS 2.0	64 hr of training over 8 days

Bryant et al. (2022) [61]	Individual	40.03, (6.95), NR	410	(a) Aged ≥18 years, (b) scores ≥16 on the K10 (c) Arabic speaking, (d) scores ≥17 on the WHODAS 2.0, and (e) had a child or dependent living in the household aged 10 to 16 years.	Group PM+	Group	Primary = HSCL-25	Facilitators (non-specialists with a bachelor's degree in a social science or a related health discipline)
Jordan	Community	73.2 % female	Psychological distress		EUC	5 weekly 2-hr group sessions	Secondary = WHODAS 2.0, PCL-5, PSYCHLOPS, PG-13, PQ-B, APQ, PMLDC	8 days of training, followed by running 2 practice groups, with weekly supervision

Chibanda et al. (2016) [62]	Cluster	35.05, (11.7), NR	573	Aged ≥18 years and resident in the area and screened positive with an SSQ-14 score of 9 or higher.	Friendship (problem-solving therapy)	Individual and group	Primary = SSQ-14 symptom score	Lay health workers (LHWs) (Mean of 10 years of education)
Zimbabwe	Primary Care	86.4% female	Anxiety/depression		EUC	6 weekly individual sessions (up to 10 weeks), plus an optional 6-session peer support program	Secondary = PHQ-9, GAD-7, WHODAS 2.0, EQ-5D	9 days training

Dawson et al. (2016) [63]	Individual	35.44, (9.78), NR	70	(a) Female, (b) aged ≥18 years and (c) scored ≥3 on GHQ-12 ≥17 or above on the WHODAS 2.0	PM+	Individual	Primary = GHQ-12	Community health workers (CHWs) (varying levels of education, no training or experience in mental health care)
Kenya	Community	100% female	Psychological distress		ETAU	Five, 90-min sessions	Secondary = WHO-DAS 2.0.; Life Events Checklist, PCL-5, WHO-VAW	8-day training program, followed by four weeks of practice cases, and then ongoing supervision

Eskici et al. (2021) [64]	Individual	36.8, (8.1), 28-50	23	(a) Aged ≥18 years, (b) being a Syrian woman with a temporary protection status, (c) speaking and understanding Arabic, and (d) score ≥1.75 on the HSCL-25	Culturally adapted CBT (CA-CBT)	Group	Primary = HSCL-25, HTQ	Arabic speaking facilitators
Turkey	Community	100% female	Psychological distress		TAU	Seven 1.5–2-hr sessions	Secondary = N/A	1 week training, with weekly (1-2 hr) supervision

Jordans et al. (2021) [65]	Cluster	44.8, (14.4), NR	611	Aged ≥18 years and could understand and speak Nepali, reporting current psychological distress and impaired functioning based on WHODAS 2.0.	Group PM+	Group	Primary = GHQ-12	NSPs (no prior mental health training, completed higher secondary school)
Nepal	Community	82.2% female	Psychological distress		EUC	5 weekly sessions lasting approximately 2.5 hours each	Secondary = PHQ-9, WHODAS 2.0, PCL, MSPSS, SSS-8	10-day training on foundational helping skills, followed by 10 days of training with subsequent supervised practice sessions

Kane et al. (2022) [66]	Individual	40.2, (9.3), NR	160	(a) Aged ≥18 years, (b) living with HIV, (c) receiving care at the clinic, (d) had unhealthy alcohol use in the past three months according to the AUDIT, and (e) met criteria for at least one comorbidity: depression, trauma symptoms, or non-alcohol substance use. A participant could also be eligible without having a comorbidity if they met criteria for a more severe alcohol use problem based on AUDIT scores.	CETA + BI	Individual	Primary = AUDIT	HIV peer educators (10–12 grade education, previously received basic training in HIV adherence counseling but with no experience or formal education in mental health or substance use therapy)
Zambia	Primary care	56% Male	Unhealthy alcohol use and depression, trauma symptoms, or substance use.		Alcohol brief intervention (BI)	1-hr weekly sessions for 6–12 sessions	Secondary = CES-D and HTQ	10 days of in-person teaching, followed by practice case

Khan et al. (2019) [67]	Cluster	Information not provided	119	Females aged ≥18 years, were deemed to be psychologically distressed, and scored ≥2 on the GHQ and ≥16 on the WHO-DAS	Group PM+	Group	Primary = HADS	Local female lay helpers (16 years of education (graduates) and with no formal training of or prior experience in mental health.)
Pakistan	Primary Care	100% female	Anxiety, depression, or PTSD		EUC	Five weekly sessions of 2 hr	Secondary = WHODAS 2.0; PHQ-9; PCL-5; PSYCHLOPS	6 days training, followed by 4 weeks of practice cases with weekly group supervision

Rahman et al. (2016) [68]	Individual	Information not provided	60	(a) Scored ≥2 GHQ-12 and (b) ≥17 on the WHODAS 2.0	PM+	Individual	Primary = GHQ-12	NSPs (no prior training or experience in mental health care delivery)
Pakistan	Primary care		Psychological distress		EUC	Five face-to-face sessions	Secondary = WHODAS 2.0, PCL-5	8 days, followed by 4 weeks supervised practice

Rahman et al. (2016) [69]	Individual	33.0, (11.8), NR	346	Scored both ≥3 on the GHQ-12 and ≥17 on the WHODAS 2.0	PM+	Individual	Primary = HADS	Lay health workers (12 to 16 years of education with no previous clinical training or experience in counseling, social work, clinical psychology, or psychiatry)
Pakistan	Primary care	78.9% female	Psychological distress		EUC	5 weekly face-to-face sessions of 90 min each	Secondary = PCL-5, PSYCHLOPS, WHODAS 2.0, PHQ-9	8 days

Rahman et al. (2019) [70]	Cluster	36.27, (9.88), NR	612	Women in the community clusters who were aged 18–60 years who intended to reside in the study catchment area for the next 6 months, and scored ≥3 on the GHQ-12 and ≥17 on the WHODAS	Group PM+	Group	Primary = HADS	Facilitators (local graduates with bachelor's degrees without mental health-care experience)
Pakistan	Community	100% female	Anxiety, depression, or PTSD		EUC	Five group sessions per week, with approximately six to eight participants per group, each session lasting for approximately 2 hr	Secondary = WHODAS 2.0; PHQ-9; PCL-5; PSYCHLOPS	7 days training

Sangraula et al. (2020) [71]	Cluster	47.9, (13.8), NR	121	Residents of the study VDCs 18 years of age and older, with a score of >2 on the GHQ-12, and a score of >16 on the WHODAS 2.0	Group PM+	Group	Primary = PHQ-9	Community-based psychosocial workers (CPSW; non-specialist psychosocial workers trained to work for NGOs)
Nepal	Community	83.5% women	Psychological distress		EUC	Five 3-hr sessions of Group PM+.	Secondary = PHQ-9, GHQ-12, WHODAS 2.0, PMPH and PCL	20-day psychosocial skills training

Shaw et al. (2019) [72]	Individual	31.8, (9.75), NR	29	(a) Aged ≥18 years (b) female, (c) residence in Malaysia, (d) a refugee or asylum-seeker, (e) Dari speaking, and (f) symptomatic for emotional distress (Refugee Health Screening score ≥12 on items 1–14) or other mental health symptoms.	CA-CBT	Group	Primary = RHS-15, HSCL-25, HTQ, MOS- SSS	Afghan therapist (completed secondary education but did not have training in mental health service provision)
Malaysia	Community and primary care	100% female	Emotional distress		Waitlist control (WLC)	8 weekly sessions	Secondary = N/A	Training on facilitation techniques, group content, and research procedures (no duration given)

Tol et al. (2020) [73]	Cluster	30.9, (10.9), NR	694	female adult refugee (aged over 18 years) from South Sudan living within study villages in Rhino Camp who: (a) is experiencing psychological distress based on attaining a score of 5 or more on the Kessler 6, and (b) can understand spoken Juba Arabic.	Self-help plus	Group workshops	Primary = K6	Local female facilitators (No formal mental health training or work experience, with Secondary Education)
Uganda	Community	100% female	Psychological distress		EUC	Prerecorded psychoeducational audio course of five weekly 2-hr sessions, delivered in workshops with 20–30 participants.	Secondary = PSYCHLOPS: PCL-6; PHQ-9; explosive anger; interethnic relationship; AAQ-II; WHODAS 2.0; WHO-5	4–5 days training, with further practice sessions
Weiss et al. (2015) [74]	Individual	42.8, (11.3), NR	149	Survivors of torture and militant attacks aged 18 years or older with a score of 36 or higher on the 29-question trauma measure	CETA	Individual	Primary = HSCL-25 and HTQ	CMHWs (non-specialists- medics or nurses who worked in rural Ministry of Health primary health care centers, and had received training in nonspecific counseling methods some years before)
Iraq	Community and primary care	69.8% male	Trauma symptoms, depression, or anxiety		WLC	8–12 weekly individual sessions of 50–60 min in length	Secondary = N/A	10-day training, with subsequent small practice groups

*Note*. AAQ-II, Acceptance and Action Questionnaire – version 2; APQ, Alabama Parenting Questionnaire-42; AUDIT, Alcohol use disorders identification test; CES-D, Center for Epidemiological Studies Depression; CSRI, Client Service Receipt Inventory; EQ-5D, EuroQOL 5D; GAD-7, General Anxiety Disorder-7; GHQ-12, General Health Questionnaire; HADS, Hospital Anxiety and Depression Scale; HSCL-25, Hopkins Symptom Checklist-25 (HSCL-25); HTQ, Harvard Trauma Questionnaire; K6, Kessler Psychological Distress Scale; MINI, Mini International Neuropsychiatric Interview; MOS-SSS, Medical outcomes study-social support survey; MSPSS, Multidimensional Scale of Perceived Social Support; PCL-6, Post-traumatic Stress Disorder (PTSD) Checklist -Civilian Version; PG-13, Prolonged Grief-13; PHQ-9, Patient Health Questionnaire-9; PMLDC, Post-migration living difficulties checklist; PMPH, Psychosocial Mental Health Problems; PQ-B, Brief Prodromal Questionnaire-16; PSYCHLOPS, Psychological Outcome Profiles; RHS-15, Refugee Health Screener-15; SSQ-14, Shona Symptom Questionnaire; SSS-8, Somatic Symptom Scale – 8; TMHS, Total Mental Health Symptoms Scale; WHO-5, World Health Organisation- Five Well-Being Index; WHO-VAW, World Health Organization's Violence Against Women study instrument; WHODAS 2.0, World Health Organization Disability Assessment Schedule 2.

## Data Availability

The data that support the findings of this study are available from the corresponding author upon request.
